# Health throughout the lifespan: The phenomenon of the inner child reflected in events during childhood experienced by older persons

**DOI:** 10.3402/qhw.v11.31486

**Published:** 2016-06-16

**Authors:** Margareta Sjöblom, Kerstin Öhrling, Maria Prellwitz, Catrine Kostenius

**Affiliations:** Department of Health Sciences, Luleå University of Technology, Luleå, Sweden

**Keywords:** Aging, health promotion, psychology

## Abstract

The aim of this study was to describe and gain more knowledge of the phenomenon of the inner child, reflected in events during childhood experienced by older persons. Thirteen older persons aged 70 to 91 years old were interviewed. A hermeneutical phenomenological analysis of the data revealed two main themes: *the inner child becomes visible* and *the inner child's presence through life*. The participants’ narratives showed that their understanding of the experiences included both positive and negative feelings, as well as ways to be creative, in which the inner child became visible. The participants’ experiences indicated that the inner child was present throughout the lifespan, was found in challenges that occurred in life, and could turn something bad into something good. However, the presence of the inner child could also be a source for development throughout life and could interfere with the person. The findings from this study point to older persons’ need to be recognized, acknowledged, and understood as a unique person living his or her own life. In addition, dimensions of well-being such as feeling safe, loved, supported, and creating space for fantasy and possibilities can be compared to the physical, mental, social, and existential dimensions of well-being found in WHO surveys and definitions of health. This calls for a holistic approach when caring for older persons.

Questions concerning the health and well-being of older persons are becoming more important in relation to the growing population of older persons (Swedish National Institute of Public Health [SNIPH], 2009). Laslett (1987) discussed the increased years of good health that many older persons experience. According to Björklund, Erlandsson, Lilja, and Gard (2015), more focus should be placed on resources for older persons that might promote health and well-being, instead of focusing on loss of function. *Health* has been defined by the World Health Organization as “a state of complete physical, mental and social well-being and not merely the absence of disease or infirmity” (WHO, 1946, p. 100), which is necessary for the individual's possibility of living a good and satisfying life (WHO 1986, 1998). *Well-being* can be referred to as a person's subjective experience of health (SOU, 2000). According to Svensson, Mårtensson, and Muhli (2012), well-being in older persons can be found in past life experiences, as well as in the present. Spiritual or existential well-being is also considered to be vital for the health of human beings (Melder, 2011). It appears that there is an emerging interest in including existential issues in caring for human beings although it can be a challenging aspect of professionals’ daily work. For example Udo, Danielson, and Melin-Johansson (2013) found that some nurses caring for patients dying of cancer integrated their personal self in their care, handling existential issues, and that they were helped by collegial support through regular sessions. Existential issues are an inevitable part of people's life, even though the way in which we deal with these issues is unique for each individual (Van Deurzen, 2014).

Past experiences can also be understood in accordance with Firman and Russel's (1994) description of the inner child as including all of the past hidden ages that have made up one's life journey. Jung is often referred to as the originator of the phenomenon of the inner child in his descriptions of archetypes, in which he argued that we may extend the individual analogy of the divine child to the life of mankind (Jung & Kerényi, 1969). Others have also used the concept of the inner child. For example, Kohut (1984) described the inner child in connection with the pains and strains of not being acknowledged. Winnicott (1987) focused on the communication between the child and the caretaker and on how being or not being acknowledged affects the inner child and hence the human being later in life. Cullberg Weston (2009) added the importance of the inner child's specific strengths that an individual brings into adulthood and knowledge about the contribution of the early years to the person we have become. Whether healthy or wounded, Firman and Russel (1994) argued that the inner child profoundly affects human beings’ overall expressions of themselves in the world.

According to Assagioli (1973), the inner child is a psychosynthesis of all ages, the transition from childhood to old age. Each developmental age is not left behind but forms one small part of all that we are. In addition, Assagioli holds that the psychosynthesis of the ages can be achieved by keeping the best aspect of each age alive. Firman and Russel (1994) also discuss the psychosynthesis of the ages and the importance of nurturing the child within. Lamagna (2011) studied the concept of self-relatedness and argued that an “internal attachment system” coordinates its activity in ways that experience, coordinates its activity in ways that best regulate the individual’s affects, thoughts, perceptions and behavior. He proceeded to explain that the implicit memories associated with these various states also govern our perceptions of the world and our ways of being in it. This conclusion is in accordance with (Siegel 1999, Siegel 2012), who argued that when people feel they have been listened to they are “feeling felt,” which is about being seen heard and understood by another – establishing an interpersonal connection of belonging and increasing the sense of well-being.

Antonovsky (1979) studied the salutogenic process using the sense of coherence to shows how generalized resistance resources can contribute to help people handle problems and difficulties in life. According to Lindström and Eriksson (2005), the salutogenic approach could have a more central position in creating a theoretical framework for health promotion research; in particular it could contribute to the solution of mental health promotion. Lindström and Eriksson (2011) argued that people should be seen as active, participating subjects shaping their lives through their action competence. This approach is similar to the empowerment concept in health promotion explained in the Ottawa Charter (1986). Rennemark and Hagberg (1997) studied the sense of coherence among the elderly in relation to their perceived history and the Eriksonian development stages. The results indicated that the more positive an individual's evaluation of his or her life history, the stronger the sense of coherence. Further they concluded that the social network was sometimes used as compensation for shortcomings in an individual's concept of self.

According to Heckhausen, Wrosch, and Schultz (2010), the challenges that individuals face as they develop from infancy into older age are selecting, adapting, and pursuing developmental and personal goals that reflect changing life course opportunities. Further, they highlighted the role of goal-setting processes in social relationships and interpersonal interactions throughout the lifespan. A similar social focus can be found in the World Health Organization's questionnaire about self-rated health and quality of life, which includes spirituality, religiousness, and personal beliefs (WHO, 2002). Therefore, studies that track trajectories over the life course could be helpful in understanding older persons’ health and well-being through childhood experiences.

According to Svensson et al. (2012), narratives create an experience of renewal *via* dialogue about what older persons experienced earlier in life through social contact and can be compared to what Ricoeur (1981) referred to as *meaningful wholeness*. George (2010) suggested that if we are to do justice to the older persons whose interests we are attempting to promote, we must pay attention to how older persons feel about their lives and their sense of well-being throughout life. Therefore, the aim of this study was to describe and gain more knowledge about the phenomenon of the inner child, as reflected in events during childhood experienced by older persons.

## Methods

According to van Manen (1990), phenomenology is a systematic attempt to uncover and describe the structure of lived experience. The frame of reference in this study is based largely on van Manen's (1990) hermeneutical phenomenological approach to lived experience research, which focuses on collecting and analyzing other human beings’ stories in order to understand a phenomenon.

### Participants and procedure

We invited Swedish-speaking senior citizens without cognitive disabilities to participate in the study. The participants were recruited through an organization for senior citizens and were contacted by the first author. An oral presentation and a letter presenting the aim of the study were given at one of the organization's member meetings. Altogether 13 persons, all of them retired, agreed to participate in the study. The participants included seven men and six women. Eleven participants were interviewed on the premises of the organization, and two participants were interviewed in their own homes. Eight participants had grown up in the city and five had grown up in the countryside. The ages of the participating women ranged from 72 to 85 years. All of them had children and grandchildren. All of the women had been employed at some point in their lives, and two of them had started an educational program when their children became older. The ages of the participating men ranged from 70 to 91 years. All of them had children and six of them had grandchildren. All of the men had started working early in life and six of them had changed occupation and/or studied later in life. All the interviews were coded to protect the identities of the participants (see below).

### Data collection

Data were collected through open-ended interviews conducted by the first author. A pilot interview was conducted to test the research questions, resulting in fewer questions. Instead of several detailed questions, the interview started with the broad question, *Can you describe significant events from your childhood that you have carried with you throughout life?* This approach made the interviews more open and left more space for the participants to choose which experiences to share. Supporting questions were asked, including the following examples: *Do you remember your feelings at the time? Has this affected you as an adult? Did you learn anything or change in any way after this event? Is there anything in what you have narrated that you have forwarded to your children?* The interviews lasted between 1 and 1.5 h and were tape recorded by the first author. The recorded interviews were transcribed verbatim by the first author and coded from A1 to A6 for the women and B1 to B7 for the men. The code list will be destroyed after the analysis is complete and the manuscript is published. No names, addresses, or identification numbers were collected. All through the analysis process the recordings were kept in a locked cupboard when not under the direct supervision of the researcher. The data will be archived for 10 years.

### Data analysis

The data analysis employed a hermeneutic-phenomenological approach that was inspired by van Manen (1990), and all of the authors were involved throughout the process described below. The analysis process included three steps: seeking meaning, thematic analysis, and interpretations with reflections. According to van Manen (1990), a holistic approach entails seeking meaning on different levels. The analysis started with reading all of the transcribed interviews to capture the significance in each interview and simultaneously seek the meaning of the collective picture that included all of the interviews. The analysis employed a back-and-forth movement between the whole and the parts, as described by van Manen (1990). The second step of the process was thematic analysis, which involved striving to determine the experiential structures that made up the participant's experiences. The textual units from the interviews were then organized into different experiences in several steps and were finally reduced to the main themes and the themes of the participant's lived experiences. The third and final step consisted of interpretation with reflection, a process that van Manen (1990) described as recovering the embodied meanings in the text in a free and insightful way.

### Ethical considerations

The persons at the organization for seniors were given oral and written information about the study, as outlined in the Helsinki Declaration (2008). In accordance with Swedish ethical law (SFS 2003:460), participation was voluntary, and informed consent was collected from the participants. The participants received information about their role in the project, their autonomy, and confidentiality. Thus the participants had the option to withdraw from the project at any time. They were also informed that no one apart from the authors of this article would have access to the collected material. In addition, the ethical committee in Umeå approved the research project before it started (2013/342-31Ö).

## Findings

The analysis revealed two main themes and six themes that captured the participants’ experiences of events during childhood, which were understood as reflecting the phenomenon of the inner child (Table I).

**Table I:** Overview of the main themes and themes

Main themes	Themes
The inner child becomes visible	Feeling safe, loved, and supported Feeling alone, scared, and sad Creating space for fantasy and possibilities
The inner child's presence through life	When something bad becomes good A source for development How I became who I am

### The inner child becomes visible

Based on the participants’ narratives, the understanding of their experiences included both positive and negative feelings, as well as ways to be creative, through which the inner child became visible.

#### Feeling safe, loved, and supported

The participants described experiences related to feelings of being safe, which were often connected to family and the structure of the older generation's inclusion in the home. Different people and situations added to the feeling of safety. Feeling safe was also connected to everyday activities that created habits or routines, such as buying milk from the milk shop and playing with dolls when the mother was weaving or reading. Safety was also connected to knowing that someone was always there to listen. The participants talked about growing up in safe surroundings with a secure atmosphere, which made them feel safe at home and during leisure time. One participant said, “I am thinking of safety, a safe upbringing in every respect. We lived in the same house as my grandparents, who always were at home and had time to listen” (A3).

The participants also shared experiences of feeling loved by a number of people and in many ways, even though times were hard and money was scarce. The participants remembered parents, relatives, and other adults as being loving, caring, and generous towards them. Siblings or other peers were also considered to be loving and empathetic. The participants shared stories of loving actions, for example, how bullies were confronted by siblings when they were being bullied or feeling a mother's love and generous caring in spite of hard work and poor circumstances. One participant said, “My father made an effort to take time off from his work so we could be together” (B7). The participants described experiences that were understood as being supported during childhood by parents, relatives, teachers, and other significant adults. Sometimes these adults became role models, and activities or situations served as inspiration that exemplified how to live a good life. For example, one participant described how “… an aunt with a handicap became a guide in how to reach my future goals, as far as studying was concerned, so I believed that I could do it” (A1). One other participant explained: “Thanks to my time as a scout, I learned how to be more outgoing and not to be afraid of strangers” (B2).

#### Feeling alone, scared, and sad

The participants talked about experiences during their childhood when they had felt alone, scared, and sad. With respect to their upbringing, the participants described how their parents did not encourage them or show them gratitude because at the time adults had the preconceived idea that these practices might spoil their children. Unfortunately, this lack of recognition was perceived by the youngsters as a feeling of being abandoned. One participant described it this way:

I remember when I was 13 years old and had to help my mother take care of the farm while my father was in the army… We had no milk machines and had to milk by hand… When my father came home, he didn't give me any compliments about running the farm, but rather criticized me for what I had done wrong… It was a trauma that I blamed my father for. (B3)

The participants described being scared. One participant remembered a funeral, as follows: “It was when my grandmother died. She was lying there in bed, and my mother lifted me up so I could see her. It was horrible and terrifying, and I hit my mother” (A2).

The participants talked about the sadness they felt as a child when their parents did not engage in their upbringing. They described experiences with formal and distant relationships with their parents. One participant said to his parents when he got older: “When I get to work I can say ‘you’ to all of them immediality [instead o ‘ma'am’ or ‘sir’], but at home I never say that” (B2). In those days, parents did not interfere in the upbringing of the children very much. The sadness experienced by the participants was reinforced by not being able to talk about feelings with their parents and by the fact that the parents did not always explain matters to their children. Children were often left alone with their fantasies and misinterpretations. One participant remembered being sad when his mother became ill and how this feeling was worsened by not being able to talk about it:

I was not allowed to tell my younger siblings… This made it difficult for me to trust people… My mother disappeared, and this wasn't natural for a child; was she dead or had she just left us for some other reason? (B1)

Some participants described a feeling of sadness connected to having to work from a very early age, sometimes instead of going to school, leaving no time for play or spending time with friends. One participant said, “When I came home from school I had to start working at once. The schoolwork came second and had to be done in the evening even if I was exhausted” (B6).

#### Creating space for fantasy and possibilities

The participants did not remember playing with their parents very often; instead, their experiences of playing with friends and siblings were more frequent. This play was understood as something that had supported them in developing their fantasy and creativity. Some of the participants described being lonely as a child without siblings and friends but finding creative solutions by playing with domestic animals, dolls, and other toys. One participant said:

I had a lot of imagination, but I don't remember playing with my mother or any other adult, but they told stories and I loved that… I also had a playmate, and one of our adventures was a landfill where we could find things. (A2)

The participants had memories from the war, during which Sweden was isolated and feared military invasion. The participants remembered role-playing about what they heard on the radio. One person's memories from role-playing were described as follows: “We played funeral and wedding and made flowers of newspaper. My mother felt uncomfortable but let us go on playing” (A5).

According to the participants’ descriptions, another way to spend time during childhood was listening to stories and thereby gaining experiences that contributed to the evolution of fantasy. The participants developed their imaginations through adults’ storytelling and were also inspired to read themselves. One participant remembered when a daughter of a neighbor came to look after them:

I have a fantastic memory of her because she could tell stories, and among other thing she told us the story of *The Children from the Frostmo Mountains* (a Swedish tale). Nobody had told stories to us children before. (B3)

One participant had memories from being a scout: “It was a bit of an adventure and we were a nice gang and stayed with the scouts until 15 years old” (B4).

### The inner child's presence through life

Our understanding of the participants’ experiences indicates that the inner child is present throughout the lifespan, is found in times of challenge during life, and can turn something bad into something good. However, the presence of the inner child could also be a source for development through life and interact with the person that the individual truly is.

#### When something bad becomes good

The participants experienced challenges during childhood, which proved to be good experiences later in life or led to something positive in the future. The participants experienced difficult circumstances at times during their upbringing, with little support from surrounding family and friends; however, some adults became good role models despite hardship in early relationships. One participant remembered:

I didn't spend much time with my father playing or reading during childhood because he was always working hard and to give in didn't exist… My father was a good role model and taught me to work hard, be careful, and accomplish what I was supposed to do. (B3)

For example, the participants experienced how fear felt as a child turned out to be an asset or strength later on in life and could serve as an inspiration for others when they felt scared and did not know how to handle a situation. One participant explained:

My mother didn't talk about feelings and did not explain things to me, which made me scared of many things when I grew up… When my children were small, I didn't want them to be afraid of things like darkness or going to the hospital and getting a shot, so I explained how everything worked and what they had to expect… This also had an impact on my work, to give good explanations for why you do certain things and in what way. (A5)

#### A source for development

The participants developed their own strength when growing up with strong or even authoritative parents, who guided them in how to cope with difficulties. In situations when their parents hindered them from doing something they wanted, their disappointment and pain could turn into an inner feeling of strength that helped in not giving up hope. One participant said, “If you are not satisfied with your situation, it is up to you to make a change. Everything is possible, even if you haven't got it all served on a silver platter” (B6).

The participants described how they gained strength in the form of curiosity and eagerness to learn new things and welcomed new ideas that did not follow the same paths as their parents. One participant said, “I learned a lot on my own and I always had seven to eight books at home from the library. My mother was very proud when I went to technical college” (B5).

#### How I became who I am

According to the participants, strong desires or interests were acquired during childhood. The participants described how they possessed a strong will or interest, which made it easier for them to overcome difficulties and challenges. One participant explained:

My mother died early, and I started studying to become a nurse, as the first one in my family to get an education. As I was alone with my children, I could manage thanks to my employment as a nurse. (A4)

One participant explained:

One of my teachers wanted me to go to high school. My parents said that we would lose our friends—a working class child could not go to high school. Later when I was an adult I got an education and worked until my retirement. (A6)

The participants described how they became the people they are today due to relationships with their parents or other significant adults. The participants felt they had had a happy childhood during which they felt loved and accepted. The participants experienced how a safe upbringing with a supporting social network had helped them to feel secure, even in difficult situations. One participant said:

I have learned from my parents to be loyal towards those around me… I think it is important to be generous and helpful… Since childhood, I felt loyalty towards those around me… This has become a guiding star for me to help people in less fortunate circumstances in Sweden, as well as in many other countries. (B5)

## Discussion of the method

The method of this study was inspired by van Manen (1990), who argued that phenomenological research is not only concerned with what it means to live a life but is also attuned towards caring and thoughtfulness. The aim of this study was to describe and gain more knowledge about the phenomenon of the inner child, as reflected by events during childhood experienced by older persons. The experiences of the participants were gathered in the spirit of van Manen (1990) in an attempt to answer questions about the essential human experiences that represent the nature of the phenomenon that in this study was interpreted as the inner child.

The problem of phenomenological inquiry is not always that we know too little about the investigated phenomenon but that we know too much (Lincoln & Guba, 1985). To enhance the confirmability of our study, we needed to handle our pre-understandings. We made our beliefs explicit in the analysis phase, discussing our different points of view, personal experiences, backgrounds, and professions (psychologist, nurse, occupational therapist, and health educator). According to Guba and Lincoln (1994), credibility is strengthened when the authors come from different backgrounds. In addition, to test the research questions, a pilot interview was conducted. According to van Manen (1990), the interview serves the specific purpose of answering the question: “What is the nature of this phenomenon as an essentially human experience?” (p. 66). This approach, which we used, strengthens the trustworthiness of a study and may help to determine whether the interview questions are suitable for obtaining rich data (Guba & Lincoln, 1994).

Kahneman (2013) argued that there is a difference between participants telling their stories in a narrative way and answering more structured questions in a survey. To capture the phenomenon of the inner child, the participants in this study were able to narrate stories about events during childhood. According to Kahneman (2013), these stories will be more predictive and tell more of the person's life and how he or she handles challenges than a survey.

One limitation of this study is that the older persons from the senior citizens’ organization who agreed to participate in this research study may have had a more positive outlook than those who decided not to accept the invitation to participate in the study. In addition, older persons who do not participate in the senior citizens’ organization may be less active than those who are involved in the organization. However, the findings show a wide range of narrative stories that include positive and negative experiences.

## Discussion of the findings

The aim of the study was to describe and gain more knowledge about the phenomenon of the inner child, as reflected in childhood events experienced by participants. The interpretation of the participants’ experiences was understood as the phenomenon of the inner child, which was illuminated in two main themes without any hierarchy: *the inner child becomes visible* and *the inner child's presence through life*.

In this study, the meaning of the inner child, as reflected in childhood events described by the participants, appears to encompass positive and negative events during childhood.

According to Assagioli (1973), the psychosynthesis of the ages involves the psychological parts of childhood, including spontaneous affection and joy, as well as uncontrolled impulsiveness and aggression. This notion is also consistent with Firman and Russel's (1994) thoughts on how the inner child, whether healthy or wounded, impacts how human beings express themselves.

The participants felt loved by parents, siblings, and relatives when they were young. The participants described positive experiences that were understood as being safe, loved, and supported. Different people and situations added to the feeling of safety, which helped the children to not be afraid of strangers but instead to trust people. Parents, relatives, and teachers supported the children by being positive role models who guided them towards future goals, resulting in feelings of loyalty, which were carried into adulthood and passed on to children and grandchildren. Similar to Winnicott's (1987) description of the inner child, the early emotional communication between the child and caretaker is essential for developing confidence and determining how the child will relate to others later in life. This finding is also consistent with Rennemark and Hagberg (1997), who argued that positive life history evaluations regarding the periods of early childhood and adolescence are associated with a strong sense of coherence. Parallel to these positive experiences, the participants in this study also described negative experiences. The participants narrated experiences that had caused them to feel alone, scared, and sad. The participants were often left alone with their thoughts and their own ideas when young and often did not have things explained to them by their parents, which sometimes led to misinterpretations. The participants also experienced their relationships with their parents as formal and distant, which led to distrust of their parents as well as other people. This finding appears to be similar to Winnicott's (1987) notion about being wounded by not being seen and Kohut's (1984) description of “the none empathic responding world” (p. 18). Kohut explains that this experience can lead to the development of a survival personality, which Winnicott (1987) calls a *false self*. The positive and negative experiences described by the participants in this study can be interpreted as expressed by Firman and Russel (1994): that the inner child profoundly affects human beings’ overall expressions of themselves in the world. Those authors also stress that both wounding and healing are a matter of empathic connection (in other words, creating health-promoting relationships).

In addition, the participants in this study remembered feeling curious, adventurous, and strong. These experiences were understood as creating spaces for fantasies and possibilities. Often, these fantasies were linked to something that the children did not quite understand or to something that they felt was frightening. According to Kaplan (2006), the world of fantasy and play can be helpful as an opportunity to escape from reality, by creating distance from what is happening in the surroundings. Hjort (1996) viewed play as a source of “joie de vivre” that has an immediate value for the child in acting based on his or her needs and desires. In addition, she argues that play creates models for the future development of the child and models for what personality to become.

According to Winnicott (1987), *good-enough-mothering* is what creates sufficient basic trust and security, with the help of a transitional object, to develop the ability of the child to represent himself in the world. Winnicott means that it is the creative locus between the inner and outer world where the child's experiences of play and fantasies can be expressed and where the child can develop as an individual. The concept of play is not only important in the life of the child but throughout the lifetime, as the inner child impacts our lives as adults (Cullberg Weston, 2009).

The participants in this study described the absence of adults in play situations. The participants did not mention what Newson and Newson (1979) argued is of importance, namely that the parent focus on the child, providing a “listening ear” and being the most important toy for the child. Similarly, Ginsburg (2007) noted the importance of maintaining strong bonds between parent and child through play and thereby promoting healthy child development. In contrast, the findings in this study show that the participants found playmates in peers and siblings, who were loving, supportive, and joyful and added to their feelings of adventure. Cutting and Dunn (2006) found that preschool children's successful communication and joint pretend play with siblings and friends were related to social cognitive skills. Their results showed the importance of cooperative pretend play, including shared affection in both child–friend and child–sibling conversations.

The participants in this study described how they developed their imaginations through adults’ storytelling and were inspired to read themselves. Bus and Ljzendoorn (1995) point out that interest in reading is not a natural phenomenon but is evoked by the pleasure of sharing a book with a parent. According to Aram and Shapiro (2012), parents have it in their power to contribute to their child's development of empathic skill by reading a book with content that can be interactive, inviting dialogue between the child and the parent. The fascination of reading was not only enjoyed during childhood but continued throughout life, promoting reading to children and grandchildren and adding to the feeling of well-being. Denham and Auerbach (1995) found that a mother's questions about emotional situations in stories predicted their children's helpfulness and concern towards others, which also meant that the children obtained a better understanding of their own emotions and well-being.

In the main theme *the inner child's presence through life*, the participants in this study described how they became who they are today due to their inner strength, strong will, or interest, which made it easier for them to overcome difficulties and challenges. The participants also described how when they were young, they learned the importance of carrying things through to the conclusion, even under difficult circumstances. This lesson was due to being presented with situations early in life in which they had to take responsibility over their own lives and change something bad into something good. This finding can be compared to Dale, Söderhamn, and Söderhamn's (2012) findings that independence and the ability to control their own lives in accordance with their own preferences were the ultimate goals required for participants to achieve health and well-being.

The participants in this study described living under very poor circumstances when young. In their experience, it was more important to help support the family by working than going to school. There was a gender difference in the participants’ experiences from childhood that showed that the girls helped the mother in the kitchen, and the boys helped the father outdoors with the animals or other tasks that were considered heavier. Phenomenological research shows that men and women have different life-worlds, which may cause gender segregation (Martinsen, Dreyer, Haahr, & Norlyk, 2013). In previous research, one can find examples of deprived children who experienced very bad conditions during childhood but still succeeded as adults in not only making themselves a future but also living healthy and prosperous lives (Lönnroth, 1990; Werner & Smith, 2001). The findings of this study show that the negative experiences that the participants carried with them from childhood had become an asset in adulthood. In connection to these hardships, the participants described how they were seen and heard by at least one person in the surrounding network. This is similar to a form of intrapersonal attunement of feeling felt suggested by Siegel (2007), which may promote well-being with physical and psychological dimensions. Firman and Russel (1994) argued that it is crucial for the development of a human being to be recognized, acknowledged, and understood. Winnicott (1987, 1988) calls this phenomenon *mirroring*. In addition, this notion can be connected to Cullberg Weston's (2009) argument that experiences from childhood that form the inner child have a profound effect on our lives as adults. According to Firman (1991), the dependent–independent paradox means that the authentic personality is not “childish” or “immature” but as a child *vis-à-vis* the deeper self. Firman and Russel (1994) argued that it is not until you can honestly be with the inner child with no criticism or pressure to change that you can connect to the authentic personality and the deeper self.

This study describes how the participants tried to counteract what they experienced as negative in their parents’ way of upbringing in relation to their own children, with respect to studies, interests, and ways of viewing things in life. The participants explained how they reflected on their experiences from childhood and made their own choices. For example, a very authoritative parent inflicted much pain for the child, but as an adult, that parent could serve as a role model for not giving in when facing challenges in life. When people manage to create consensus in their lives, they also achieve high narrative competence, which increases mental health (Havnesköld and Risholm Mothander, 1995). In addition, this process of making one's own choices can be compared to Heckhausen et al. (2010), who argued for the importance of goal-setting processes in social relationships and interpersonal interactions throughout the lifespan.

The participants gained strength from their curiosity to learn and welcome new ideas that did not always follow the same paths as their parents. The participants also experienced how their own strength became a source for development, helping them to not give up hope when they were hindered in fulfilling future aspirations. According to Clancy, Balteskard, Perander, and Mahler (2015), recollecting past experiences and sharing stories can create an orientation towards the future and be important for hope and a feeling of possibilities. The participants experienced how a safe upbringing with a supporting social network had helped them feel secure even in difficult situations. They had had a happy childhood when they felt loved and accepted. This finding is in accordance with the findings of Svensson et al. (2012), who emphasized the importance of factors such as well-being and the maintenance of a social network for experiencing a good quality of life.

The results of this study show that the phenomenon of the inner child is reflected in events during childhood and that these experiences are remembered throughout life and relate to the well-being of older persons. This is in some way what Tornstam (2011) talks about: a reconciling of experiences and memories and the idea that we are all ages at the same time. However, his conclusions that older persons become less interested in social interactions and have a greater need for solitary meditation were not noted in this study. Instead, the findings of this study point in a somewhat complex direction, showing that some older persons became more contented, while others were weighed down by remembering their childhood experiences throughout life, which affected their interest in being socially active. According to Berg, Hassing, McClearn, and Johansson, McClearn, and Johansson (2006, 2009), social network quality, a sense of being in control of one's life, and decreased depressive symptoms were significantly associated with higher life satisfaction among the oldest individuals. According to Antonovsky (1979), a sense of coherence helps people handle problems and difficulties in life as some people stay healthy and develop a sense of meaning in their lives despite stressful and traumatic experiences. Even if he does not mention the inner child, Antonovsky (1979), talks about resistance resources as a sort of inner strength. However, he does not connect the resistance resources as strongly to the relation between the human being and his or her social network during childhood, which was described by the participants in this study.

In conclusion, the findings of this study demonstrate new knowledge illuminating how human beings are influenced by the inner child throughout their lifespan. Experiences during childhood have an impact on the choice of profession and also on how we act in relation to the next generation. The participants in this study voiced a need to be recognized, acknowledged, and understood as unique persons living their own lives. This finding is consistent with Assagioli's (1973) argument that the inner child is a psychosynthesis of all ages and that the psychosynthesis of the ages can be achieved by keeping the best aspect of each age alive. In accordance with George (2010), we argue that attention should be paid to how older persons feel about their lives, as well as to the strategies that they need to sustain a sense of well-being. In other words, when caring for older persons keeping Firman and Russel's (1994) notion of nurturing the inner child in mind can be of great value for promoting health. The findings of this study point to the well-being dimensions of feeling safe, loved, and supported, as well as creating space for fantasy and possibilities. These dimensions can be compared to the physical, mental, social, and existential well-being dimensions that are found in the WHO's definitions of health (WHO, 1946, 1986, 1998) and surveys (WHO, 2002). Although the sample in this study was limited, we believe the findings are worthy of recognition as we suggest that a holistic approach be used when caring for older persons.
